# The Natural History of Spina Bifida in Children Pilot Project: Research Protocol

**DOI:** 10.2196/resprot.2209

**Published:** 2013-01-25

**Authors:** Ann I Alriksson-Schmidt, Judy K Thibadeau, Mark E Swanson, David Marcus, Kari L Carris, Csaba Siffel, Elisabeth Ward

**Affiliations:** ^1^Musculoskeletal Sciences, Department of OrthopedicsClinical Sciences LundLund UniversityLundSweden; ^2^Centers for Disease Control and PreventionAtlanta, GAUnited States; ^3^Children's Healthcare of AtlantaDepartment of NeuropsychologyAtlanta, GAUnited States; ^4^NORC at the University of ChicagoChicago, ILUnited States; ^5^Carter Consulting, Inc., Consultant to CDCAtlanta, GAUnited States

**Keywords:** spina bifida, musculoskeletal disorder, health, children, follow-up

## Abstract

**Background:**

Population-based empirical information to inform health care professionals working with children with spina bifida currently is lacking. Spina bifida is a highly complex condition that not only affects mobility but many additional aspects of life. We have developed a pilot project that focuses on a broad range of domains: surgeries, development and learning, nutrition and physical growth, mobility and functioning, general health, and family demographics. Specifically, we will: (1) explore the feasibility of identifying and recruiting participants using different recruitment sources, (2) test a multidisciplinary module to collect the data, (3) determine the utility of different methods of retrieving the data, and (4) summarize descriptive information on living with spina bifida.

**Objective:**

The overall objective of the project was to provide information for a future multistate prospective study on the natural history of spina bifida.

**Methods:**

Families with a child 3 to 6 years of age with a diagnosis of spina bifida were eligible for enrollment. Eligible families were identified through a US population-based tracking system for birth defects and from a local spina bifida clinic.

**Results:**

This is an ongoing project with first results expected in 2013.

**Conclusions:**

This project, and the planned multistate follow-up project, will provide information both to health care professionals experienced in providing care to patients with spina bifida, and to those who have yet to work with this population. The long-term purpose of this project is to increase the knowledge about growing up with spina bifida and to guide health care practices by prospectively studying a cohort of children born with this condition.

## Introduction

### Spina Bifida Overall

Spina bifida (SB) is a neural tube defect (NTD) that occurs early after conception when the neural tube that forms the brain and the spine does not close properly. SB generally is considered one of the most complex birth defects compatible with life [1]. In recent decades, survival rates have increased dramatically, primarily due to improved care and the use of antibiotics [2]. A recent study suggested that the overall prevalence of SB among children and adolescents in 10 regions of the United States was 3.1 per 10,000 in 2002 [3]. Although rates of SB differ substantially across countries [4], probably no country is excluded from having children born with this potentially disabling condition. The necessary follow-up surgery and care can present considerable physical, emotional, and financial burdens. In spite of substantial differences between and within countries in terms of care available and provided to individuals with SB, it is important to study how SB impacts the child and his/her family long-term.

The most severe form of SB, myelomeningocele, is the most common of the NTDs, and the most complex birth defect compatible with long-term survival [5]. There are a substantial number of people whose everyday lives are directly or indirectly affected by SB, prompting a real need for prospective comprehensive data to provide evidence regarding health promotion, prevention of secondary conditions, access to appropriate preventive health care, and caregiver support. We know of no US—and few international—population-based studies or programs focusing on the natural history of SB. This is important because people with SB often experience condition-specific difficulties (eg, incontinence, mobility limitations, and cognitive challenges) and secondary conditions (eg, pressure sores, urinary tract infections, and depression) that detrimentally affect several aspects of their lives. Little information is known about how and when to intervene to prevent or reduce the number of modifiable problems from occurring during childhood, and there is still much to be learned about the natural course of SB throughout the lifespan. Because SB is a relatively rare condition and people with SB traditionally have not lived to adulthood, many—if not most—available treatments are based on expert opinions in lieu of evidence-based research [6]. To remedy this lack of data, the current pilot project was developed by the National Spina Bifida Program at the Centers for Disease Control and Prevention (CDC) in collaboration with partners at NORC (formerly the National Opinion Research Center) at the University of Chicago, and the Neuropsychology Department at Children’s Healthcare of Atlanta. The current project will provide useful information on research design and methodology that will inform the planning and implementation of a prospective US multistate SB project. The larger prospective study will enroll a large cohort of children with SB to provide population-based data on some of the most pressing concerns for this population.

Part of the complexity associated with SB is that a number of body systems tend to be severely affected. In this project, we focus on the following medical specialties: orthopedics (eg, mobility), urology (eg, incontinence, urinary tract infections, and renal failure), and neurosurgery (eg, hydrocephalus and Arnold-Chiari II malformation). Psychosocial issues and specific learning problems also are reported frequently and addressed in the project. A brief review is provided in the following sections.

### Medical Concerns, Mobility, and Functioning

SB is challenging—it affects neurological functions, urological and kidney functions, and mobility for virtually everyone with the condition. Secondary conditions such as pressure sores [7] and pain [8] are other areas of concern. Children with SB often undergo multiple surgeries and need to adhere to long-term medical and behavioral treatments. Hydrocephalus co-occurs with SB 80-95% of the time [9-12] and most of these children exhibit Arnold-Chiari II malformation [11]. These brain abnormalities typically require neurosurgical interventions in the form of shunt insertions, shunt revisions, decompression, or any combination thereof. The presence of hydrocephalus and Arnold-Chiari II malformation has been associated with worse performance on certain cognitive tasks [11]. Shunt revisions also have been associated with negative outcomes. Results from a British community-based follow-up study of adults with SB showed an inverse relationship between the number of shunt revisions and long-term achievement as defined by level of independence, using a car, and employment [13].

Other medical consequences include neuropathic bladder, malfunctioning kidneys, urinary tract infections, and urinary and fecal incontinence. Although overall urological goals are similar if not identical (in that they focus on maintaining normal renal function, gaining urinary continence, and maximizing independence [14]), the methods used to achieve these goals differ among health care providers. Renal failure still is reported as a leading cause of mortality and morbidity among people with SB [15-16], even though renal failure among this group is almost completely preventable [17]. Incontinence occurs frequently, can interfere with achieving independence, and is a source of embarrassment for the individual [18]. Estimates of how often incontinence occurs, and to what extent it affects life, differ depending on the definition of incontinence, as well as sampling methods. Among young adults in Europe with SB, approximately 60% reported being incontinent, regardless of the type of bladder management used, and approximately 70% reported that being incontinent presented a problem [18]. Clean intermittent catheterization (CIC), medication, and surgeries are typical methods of treating incontinence. Many urologists recommend that CIC start at an early age [14,17], but research is needed on the timing and method of implementation of a CIC plan, as are prospective data on how a successful CIC regimen can be achieved. A French study assessing the frequency and types of associated malformations of NTDs showed that about 25% of infants with SB had at least one other major malformation, including orofacial clefts and cardiac defects [19]. These malformations require medical interventions and could further complicate the lives of people with SB.

Mobility is affected negatively among most people with SB and is related to the level of lesion (LOL). Different definitions of LOL exist, but generally a higher LOL results in more severe mobility restrictions. The presence of scoliosis, kyphosis, club foot, hip and knee contractures, or other orthopedic conditions habitually warrant surgery and can affect mobility, and subsequently independence, negatively.

### Development and Learning

There is great heterogeneity in terms of cognitive function among people with SB [20]. In addition to the SB diagnosis, performance on cognitive assessments is contingent on factors such as having a higher LOL, hydrocephalus or shunting, or both, and Arnold-Chiari II malformation [11,21-23]. Researchers from an Australian population-based study linked several databases from Western Australia and reported that 18.8% of people born with SB (1980-1999) had received a diagnosis of intellectual disability (IQ <70) during childhood. In comparison, only 1% of individuals born during the same period but without a diagnosis of a birth defect had a diagnosis of intellectual disability [24].

Regardless of overall cognitive function, people with SB are at increased risk of specific problems that adversely affect their ability to learn, often resulting in academic difficulties. Substandard scores on certain types of memory tests [9,25] and a number of tasks related to executive functions [12,26] are reported consistently. In addition, attention-deficit/hyperactivity disorder, and in particular the predominantly inattentive type, has been reported more frequently among this population compared with their peers without SB [10]. Learning problems, independent of overall cognitive ability, often make up an additional burden for people living with SB. Studies generally have suggested that nonverbal learning problems (eg, deficits in motor, visual-spatial, and mathematical abilities) in particular constitute an area of concern. Although the increased risk of learning problems among people with SB has been established, there is a need for prospective research that assesses early predictors of learning problems and development, as well as research on strategies that can facilitate functioning for people with these types of problems.

### Physical Growth, Nutrition, and General Health

While parts of the world struggle with lack of a steady food supply and malnutrition, obesity has received much attention in the United States and parts of Europe. Lack of exercise and unhealthy eating habits have long been associated with preventable morbidity and preterm mortality. Few researchers have focused on weight issues among people with SB. However, it is known that, in general, individuals with disabilities in the United States are at higher risk than people without disabilities of not reaching recommended levels of exercise [27] and are at an increased risk for obesity and poor health. Many reasons for not exercising are similar for people with and those without disabilities, although people with disabilities can face additional hurdles to exercising that are unique to them. Barriers in the environment have been listed as an important correlate of lack of exercise for people with disabilities [28]. In a Japanese study that addressed weight among children with SB, no significant differences in percentage of body fat were found between a group of children with SB and a control group without SB before 5 years of age. An increase in body fat was noted among the group with SB after 6 years of age [29]. Additionally, a positive relationship between the presence of hydrocephalus and a higher percentage of body fat was noted [29].

The overall aim of the study was to assess the research design and methodology to inform a future multistate prospective study on the natural history of SB. In addition, the project has four main objectives: (1) to explore and compare the feasibility of identifying and recruiting participants using different recruitment sources, (2) to test a multidisciplinary model to collect data, (3) to determine the utility of different methods of retrieving data (ie, telephone surveys, in-person assessments, and record abstraction), and (4) to summarize preliminary descriptive information on the natural history of SB. As part of the third objective, we will investigate whether participants prefer in-person assessments or a telephone survey.

## Methods

### Participants

**Figure 1:**
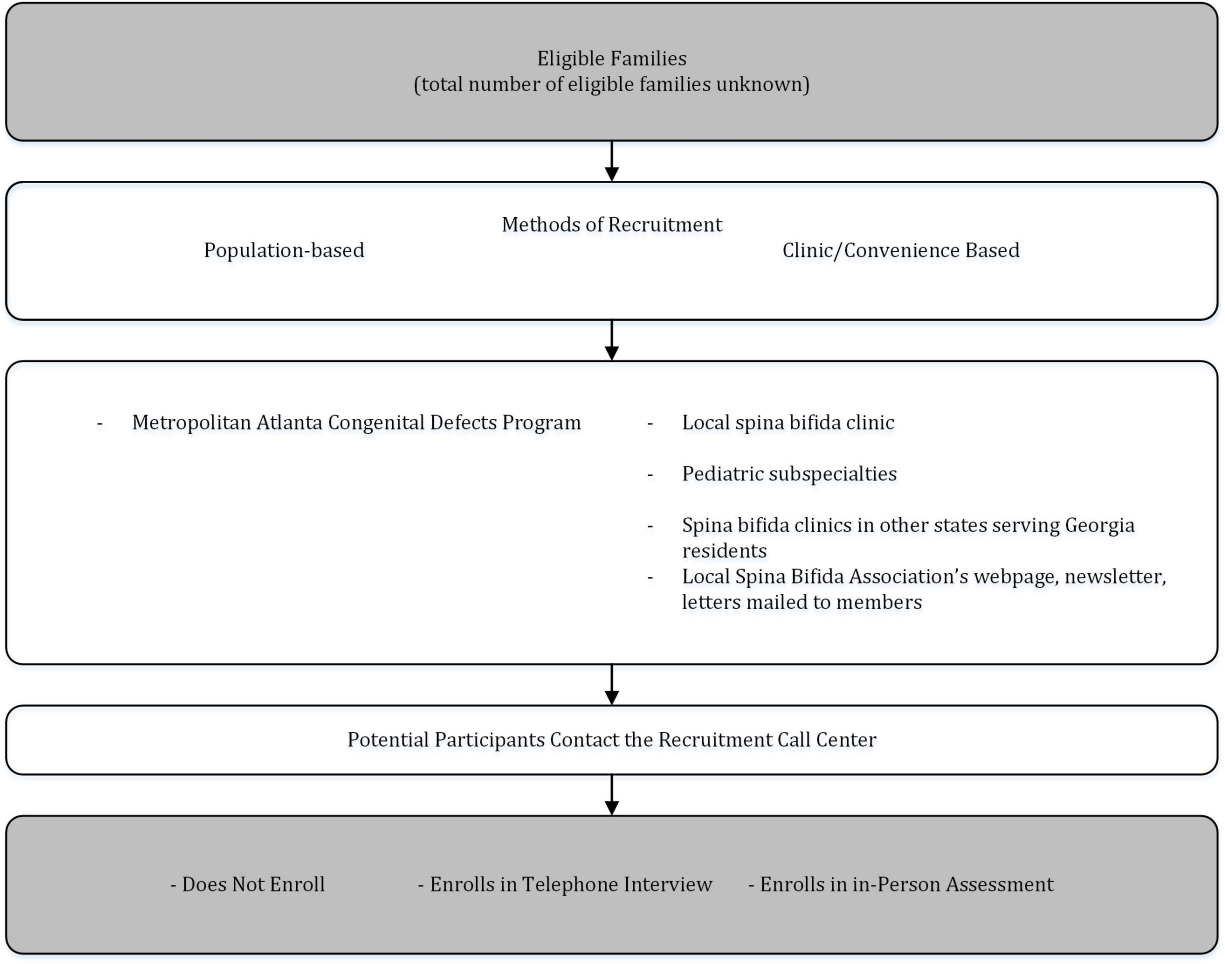
Recruitment Scheme.

Families with a 3-, 4-, 5-, or 6-year-old child with a diagnosis of SB (International Statistical Classification of Diseases and Related Health Problems, Ninth Revision, Clinical Modification (ICD-9-CM) codes 741.0 and 741.9 without 740.0 and 740.1 or the codes 741.00-741.99 without 740.00-740.10 from the modified British Paediatric Association coding system) currently residing in the State of Georgia in the United States will be eligible to participate. Confirmation of diagnoses will be possible in those cases where we can extract the ICD code from the medical records. Children with a diagnosis of SB occulta will be excluded, as the natural history of SB occulta is presumed to be quite different from that of SB aperta. CDC already tracks children born with certain congenital conditions, including SB, in selected areas in a limited number of states in the United States. In Georgia, this population-based surveillance system is called the Metropolitan Atlanta Congenital Defects Program (MACDP). Using MACDP for recruitment purposes provides certain strengths for the project, such as an established sample frame of eligible children, a physician-confirmed diagnosis of SB, and limited contact information that is updated periodically. A drawback of recruiting solely from the MACDP is that it is limited to five metropolitan Atlanta counties. The experience of growing up with SB might be quite different for children with SB and their families who are not living in a metropolitan area. For example, the type of care available and the experience and knowledge of the professionals working with these children and their families might differ. Recruitment strategies for these families will be different as well. Therefore, a convenience sample will be included and we will use different methods to recruit families who are not part of MACDP (Figure 1). The convenience sample also will provide information on how to create a sampling frame in the absence of a surveillance program.

### Data Collection

Family involvement will be solicited in the SB clinic in Atlanta in person and by mail, or through a letter from the MACDP. A recruitment center headed by NORC will be notified of those who are interested and those families will be contacted. After an interested family is contacted by the recruitment center, the project recruiter will ask the parent to orally confirm that the child has a diagnosis of SB (myelomeningocele) and is in the 3–6 years of age range. An explanation of the project will follow, during which parents will be encouraged to ask any project-related questions they might have. If they are interested in participating, contact information will be obtained. Next, the parents will choose which project component they wish to complete (telephone survey or in-person assessments). Only the telephone component is available for monolingual Spanish-speaking individuals. Randomizing participants into project components would be desirable. However, because we are interested in assessing which component parents prefer, and because long driving distances might deter those living far from the assessment site from participating if they were to be randomized to the in-person component, we decided not to randomize. If a parent chooses the in-person component, an appointment will be scheduled and a reminder letter and directions to the assessment site will be mailed to the parent. If a parent chooses the telephone survey component, the project recruiter will proceed either by conducting the survey or by scheduling an appointment to complete the survey at a later time. Parents who choose not to participate will be asked for the main reason for that decision (open-ended) and thanked for having taken the time to learn more about the project. A separate recruitment log will be used to record the reasons stated for deciding not to participate. These data will be an important part of the project, as anecdotal information has shown it is difficult to recruit participants for research related to SB. These data can provide direction on how to design future longitudinal projects to maximize participation.

### Procedure

#### Telephone Survey Component

After oral consent is obtained, the interviewer will read all questions verbatim in the order indicated in the questionnaire. Each participant’s responses will be marked directly on the paper-and-pencil interview copy of the survey. At the conclusion of the telephone survey, the interviewer will store the completed paper-and-pencil interview form in a secure, locked cabinet and project staff will enter the survey data into an electronic data file that will be stored on a secure network. Identifying information that is collected during the course of the telephone survey (eg, names, addresses, and telephone numbers) will be separated physically and permanently from the survey data and entered into a separate database. The telephone survey is estimated to last approximately 90 minutes. Participants will receive $25 for participating.

#### In-Person Component

Data will be collected from both the child and the parent. After the informed consent process is completed, a licensed clinical child neuropsychologist will assess the child. The parent will complete the parent portion of the assessment in a separate room with assistance from the project coordinator (Table 1). Assessments and questionnaires that require scoring will be scored immediately after the participant has completed the entire project component (Table 2). The in-person component is estimated to last no more than 3 hours per family. To reduce the time commitment of the in-person component, the child and parent will be assessed or interviewed, as applicable, simultaneously when possible. Each family will receive a $50 honorarium and get reimbursed for transportation costs.

The project survey, created specifically for this project, contains items related to medical concerns, development and learning, nutrition and physical growth, mobility and functioning, general health, and family demographics. The survey used in the in-person component contains the same items as the survey used in the telephone survey component. Parents participating in the in-person component will be asked to fill out the McMaster Family Assessment Device which measures family functioning, the Behavior Rating Inventory of Executive Function- Preschool version (BRIEF-P) to measure executive functioning, and the Pediatric Evaluation of Disability Inventory (PEDI) which measures functional abilities. Parents will also be asked to fill out the Adaptive Behavior Assessment System (ABAS), which measures daily living skills, and the Children's Health Care Patient History Questionnaire, developed by the neuropsychologists at the Children’s Healthcare of Atlanta clinic. The neuropsychological battery will consist of 5 separate assessments: the Differential Abilities Scale II (DAS-II) will be used to measure cognitive abilities, the Peabody Picture Vocabulary Test 4^th^ edition (PPVT-4) will be used to measure receptive vocabulary, the NEPSY-II will be used to measure cognitive abilities, the Wide Range Assessment of Visual Motor Abilities (WRAVMA) will be used to measure visual-motor integration, and finally, the Bracken Basic Concept Scale (BBCS-R) will be used to measure basic concept acquisition, receptive language, and school readiness. More details on the different study instruments can be found in Tables 1 and 2.

#### Medical Records and Early Intervention Data Collection

If the parent completes the telephone survey, he or she will be mailed hardcopy forms to authorize release of the child’s medical and early intervention records. The parent will be asked to read, sign, and return the forms in a self-addressed, stamped envelope provided by the project. If the parent completes the in-person component, written authorization to release the child’s medical and early intervention records will be sought at the beginning of the in-person session. Copies will be made of the medical records or the early intervention records, or both, at the respective clinics and sites and taken to the coordinating office. Relevant data will be extracted from the records and transferred onto 2 separate forms that have been created specifically for the project. Reliability is often a concern in most types of records abstractions. In this pilot, we are primarily interested in investigating if the data we are interested in can be found in the records.

### Data Analysis

Descriptive statistics will be computed on the quantitative data. Specifically, means, standard deviations, and confidence intervals will be computed for continuous variables, and frequencies and percentages will be computed for dichotomous and categorical data. If the sample size is sufficient, we will compare the participants based on LOL, sex, and race and ethnicity using multivariate statistics. The child assessment results will provide information on how this sample of young children with SB scored compared with the normative scores. We also will reevaluate whether the standardized measurements and tests were appropriate for this specific group of individuals, or whether other measurements might be more appropriate in the future. The qualitative data will be reviewed carefully, summarized, and used to inform future recruitment of individuals with SB or other potentially disabling conditions, with an emphasis on improving recruitment strategies for surveillance systems. Following project completion, recruitment data will be reviewed and summarized. We also will calculate how many participants chose the in-person component versus the telephone survey component. Participant feedback on both components will be reviewed carefully and summarized to guide and inform potential changes that might be necessary to improve future projects. Missing data patterns will be reviewed to assess if there were particular items or sections that families were more likely to skip. These types of data are essential, as the long-term goal is to follow children with SB longitudinally.

### Ethical Considerations

The project already has undergone ethical review and been approved by three separate institutional review boards (US government, university, and hospital). The project has also undergone Office Management and Budget review and been approved. Oral consent will be obtained from those participating in the telephone survey and written consent will be obtained from those participating in the in-person component. Verbal assent will be obtained from children 6 years of age before they participate in the in-person component.

### Limitations

As in many studies that do not rely solely on clinic-based samples, we have no accurate way to determine how many families are eligible to participate, thus making it difficult to make an assumption of sample size. We will use different approaches to inform and recruit participants. Although the current project is cross-sectional, preventing determination of causality, we are in the process of planning a multistate prospective study that will be better suited to address causality, as appropriate. The in-person component will not be available in Spanish, which will limit the options for monolingual Spanish-speaking participants.

**Table 1:** Parent administered instruments to be used.

Instrument	Number of items	Topics/domains	Cronbach alpha coefficients	Reliability coefficients
Project survey^a,b^	201	(1) medical concerns, (2) development & learning, (3) nutrition & physical growth, (4) mobility & functioning, (5) general health, & (6) family demographics	not applicable	not applicable
McMaster Family Assessment Device [30]^b^	60	Family functioning (1) problem-solving, (2) communication, (3) roles, (4) affective responsiveness, (5) behavior control, & (6) general functioning	.57-.86 [31]	one-week test-retest .67-.76 [31]
BRIEF-P [32]^b^	63	Executive functioning Subscales (1) emotional control, (2) shift, (3) inhibit, (4) working memory, & (5) plan/organize Indices (1) inhibitory self-control, (2) flexibility, & (3) emergent metacognition	.80-.95 [32]	4.5-week test-retest- .78-.90 [32]
PEDI [33]^b^	217	Functional abilities Subdomains (1) self-care, (2) mobility, & (3) social function Parts (1) functional skills, (2) caregiver assistance, & (3) modifications	.95-.99 [33]	not applicable
ABAS-II [34]^b^	241	Daily living skills 10 skill areas Domains (1) social, (2) practical, & (3) conceptual	.98-.99 [34]	.90 [34]
Children’s Health care Patient History Questionnaire^b^		6 sections (1) identifying information, (2) pregnancy & newborn history, (3) developmental history, (4) medical history, (5) educational background, & (6) social history	not applicable	not applicable
Medical & early intervention records data abstraction forms^a,b^		For medical and early intervention abstraction from records Medical (1) neurosurgery, (2) urology, (3) orthopedics, & (4) hospitalization	not applicable	not applicable

^a^Used in the telephone component

^b^Used in the in-person component

**Table 2:** The child assessments to be administered (in-person component only).

Assessment	Topics/domains subtests included	Cronbach alpha coefficients	Reliability coefficients
DAS-2 [35]	Cognitive abilities, 7 core subtests from early years battery (1) verbal comprehension, (2) picture similarities, (3) naming vocabulary, (4) recall of objects, (5) pattern construction, (6) matrices, & (7) copying	not applicable	not applicable
PPVT-4 [36]	Receptive vocabulary 20 content areas and parts of speech across all levels of difficulty	.94 [36]	one-week test-retest-.93 [36]
NEPSY-II [37]	Cognitive abilities (1) comprehension of instructions, (2) word generation, & (3) sentence repetition	.90-.91[37]	.72-.89 [37]
WRAVMA [38]	Visual-motor integration WRAVMA matching visual-spatial subtest WRAVMA pegboard fine-motor subtest	exceeding .90 [38]	.81-.91 [38]
BBCS-R [39]	Basic concept acquisition & receptive language, school readiness composite (1) colors, (2) letters, (3) numbers/counting, (4) sizes, (5) comparisons, & (6) shapes	not applicable	.86 [39]

## Discussion

The lack of information about the natural history of SB needs to be rectified by collecting multistate longitudinal data at all life stages. Having this information will facilitate the development of appropriate health care recommendations and general guidelines for people with SB at different life stages, affecting outcomes in self-management, relationships, and learning and employment. It is imperative that knowledge be gained regarding the identification of developmental delays before the optimal time of developmental achievement has passed. Determining the interventions needed to address these delays will help people living with SB to be more likely to realize their full potential. Such an undertaking requires pilot testing of the proposed methods prior to implementation on a larger scale. The current project, although cross-sectional, is a first step towards recruiting and following a larger sample of children born with SB. By using different data retrieval methods, we will learn which yield the most valid and reliable data. We also will learn what data collection method is most acceptable to participating families, optimizing participation rates and reducing attrition for future projects.

## Abbreviations

ABAS-II: Adaptive Behavior Assessment System
BBCS-R: Bracken Basic Concept Scale
BRIEF-P: Behavior Rating Inventory Preschool Version
CDC: Centers for Disease Control and Prevention
CIC: clean intermittent catheterization
DAS-2: differential abilities scale 2
ICD: International Statistical Classification of Diseases and Related Health Problems
LOL: level of lesion
MACDP: Metropolitan Atlanta Congenital Defects Program
NEPSY-II: developmental neuropsychological assessment
NTD: neural tube defect
PEDI: Pediatric Evaluation of Disability Inventory
PPVT-4: Peabody Picture Vocabulary Test
SB: spina bifida
WRAVMA: Wide Range Assessment of Visual Motor Abilities
